# Functional Analysis Decision-Making Considerations

**DOI:** 10.1007/s40617-025-01057-w

**Published:** 2025-04-09

**Authors:** Katherine R. Brown, Casey Irwin Helvey, Michael P. Kranak, Abigail Lavin

**Affiliations:** 1 Psychology Department, Utah State University’s Sorenson Center for Clinical Excellence, 6405 Old Main Hill, Logan, UT 84321 USA; 2 Rutgers Robert Wood Johnson Medical School, New Brunswick, NJ USA; 3 Children’S Specialized Hospital - Rutgers University Center for Autism Research, Education, and Services (CSH-RUCARES), New Brunswick, NJ USA; 4 Oakland University, Rochester, MI USA; 5 Oakland University Center for Autism, Rochester, MI USA

**Keywords:** Assessment, Challenging behavior, Clinical decision-making, Functional analysis methodology

## Abstract

Functional analysis (FA) is often considered an integral component of treating severe challenging behavior (e.g., aggression, self-injury). Given its essential nature, there are a growing number of publications aimed at supporting clinicians’ understanding of FA design and methodological refinements for addressing barriers to implementation. The current paper builds on previous work by offering a guide for clinicians new to FA implementation. In particular, guidance is provided on *when* and *under what conditions* to include specific FA test conditions and how to select a given methodology for a case. In addition, we provide an in-depth discussion on best practices for safe and ethical FAs, regardless of the chosen setting, implementer, or methodology.

## Functional Analysis Decision-Making Considerations

Functional analysis (FA) is a well-established empirical assessment that informs function-based interventions to decrease challenging behavior and increase appropriate behavior. Since the inception of the experimental FA (Iwata et al., 1982/1994), researchers have continued to extend and refine FA-related methodology and procedures. To encourage the widespread use of FA and support valid FA processes, researchers have published practice guidelines to support clinicians in selecting, designing, and implementing effective FAs. For example, Iwata and Dozier (2008) developed a tutorial for clinicians new to FA methodology that reviewed experimental designs and data analysis interpretation. Hanley (2012) developed a practice guide on how clinicians can overcome known implementation barriers, such as low-rate challenging behavior, covert challenging behavior, and potentially dangerous behavior.

With FA implementation becoming increasingly common in clinical settings (Melanson & Fahmie, 2023), there is a growing number of publications aimed at supporting clinicians’ understanding of FA experimental designs, refinements to FA methodology that address a wide range of previously identified barriers to FA implementation, and how to design relevant FA test and control conditions (e.g., see Dozier et al., 2022; Falligant et al., 2023). As such, we will not be reviewing this information and encourage unfamiliar readers to review these publications. This manuscript aims to guide clinicians new to implementing FAs in clinical practice on *when* and *under what conditions* they should include specific FA test conditions and select a given FA methodology for a client. This is important because in order for behavior analysts to best serve clients who engage in challenging behavior, they need to learn multiple FA methodologies and be able to discriminate the conditions under which one should be selected relative to another (e.g., multielement, trial-based; Haymes et al., 2024; Lambert et al., 2022). To do this, clinicians must attend to the experimental design (e.g., multielement, reversal) and FA method (e.g., traditional, trial-based, precursor) and select each based on specific clinical variables rather than defaulting to one design method in *all* cases. In addition, we provide an in-depth discussion on best practices for safe and ethical FAs, regardless of the chosen setting, implementer, or methodology. To aid in understanding key concepts and terms used throughout this paper related to FA, Table 1 is a glossary of relevant terms that we encourage readers to reference.

**Table 1:** Glossary of functional analysis terms

Term	Definition
Accuracy	The identification of “true” reinforcers maintaining challenging behavior ^a^
Control Condition	A condition in which access to reinforcement is freely given and not contingent upon any behavior ^b^
Differentiated	When using structured criteria, challenging behavior reliably occurs in or more test conditions and is separated from the control condition and/or responding in challenging behavior meets classification for automatically maintained behavior ^c^
Descriptive Assessment	The use of direct observation and measurement of challenging behavior, without manipulation of variables, to generate hypotheses on behavior function ^d^
Discriminant Validity	The ability of an assessment to distinguish between different reinforcer classes ^a^
Ecological Validity	The extent to which a procedure corresponds with real-word settings and phenomena ^e^
Establishing Operation	A condition that both increases the value of a reinforcer and increases the rate of responding to gain that reinforcer ^f^
Experimental Design	A single-subject design that allows for observation of behavior under test and control conditions such that causal relationships can be identified ^g^
External Validity	The extent to which observed results are consistent outside clinical settings and implementers ^h^
False Negative	Failure to identify a reinforcer that maintains challenging behavior ^a^
False Positive	Identification of a reinforcer that does not maintain challenging behavior ^a^
Functional Analysis	An empirical demonstration of causal relationships between sources of reinforcement and challenging behavior ^g^
Iatrogenic Effects	A nonfunctional, but highly preferred stimulus induces a novel function when combined with a functional reinforcer ^i^
Indirect Assessment	An open-ended or structured interview of clients and/or caregivers who have previously witnessed the target challenging behavior ^d^
Interactive Effect	The interaction of two or more reinforcers acting on a behavior to produce outcomes that would not occur in the presence of a single reinforcer alone ^j^
Internal Validity	The extent to which results are due to a causal relationship between variables ^h^
Methodology	Methodological characteristics that distinguish different types of functional analysis ^k^
Predictive Validity	Ability of a functional analysis to predict treatments that will or will not reduce challenging behavior ^a^
Sensitivity	The ability of functional analysis to identify reinforcers maintaining challenging behavior ^a^
Specificity	The ability of functional analysis to rule out reinforcers that do not maintain challenging behavior ^a^
Test Condition	The condition in which a reinforcer is delivered contingent on challenging behavior ^b^
Undifferentiated	If challenging behavior does not meet structured criteria resulting in the identification of at least one function or never occurs during the functional analysis ^c^

^a^ Tiger and Effertz (2021). ^b^ Hanley (2012). ^c^ Weber et al. (2024). ^d^ Tarbox et al. (2009). ^e^ Fahmie et al., (2023). ^f^ Strohmeier et al. (2017). ^g^ Iwata and Dozier (2008). ^h^ Shadish et al. (2002). ^i^ Retzlaff et al. (2020). ^j^ Fisher et al. (2016). ^k^ Melanson and Fahmie (2023)

## Conditions Under Which to Conduct an FA

Although FA implementation is becoming more common in clinical settings (Melanson & Fahmie, 2023), surveys of practicing BCBAs continue to report a sizeable research-to-practice gap, with the minority of BCBAs reporting implementation of FAs to inform intervention (Oliver et al., 2015). This is problematic given that FAs allow clinicians to develop more effective treatments (Campbell, 2003; Herzinger & Campbell, 2007; Heyvaert et al., 2010, 2014) and decrease reliance on punishment procedures and pharmacological interventions to reduce challenging behavior (Axelrod, 1987; Kahng et al., 2002; Mace et al., 1991). In addition, clinicians are ethically obligated to use evidence-based assessments to inform treatment development (Behavior Analyst Certification Board, 2020). Moreover, not implementing an FA can create delays to effective intervention, which can have several deleterious effects, including injury to the client or others, significant damage to the environment, and loss of caregiver buy-in or rapport. These are just some of the many risks of not conducting an FA to inform a function-based intervention.

Implementing an FA is particularly important in cases in which challenging behavior is unsafe, results in moderate-to-significant physical injury, or is life-threatening. As such, clinicians should carefully consider the topography, intensity, and frequency of challenging behavior when considering whether to implement an FA in lieu of using less rigorous methods to inform intervention (i.e., functional behavior assessment methods). For example, if a client engages in challenging behavior in the form of dropping to the ground that occurs approximately twice per week and results in no injury. In that case, clinicians may consider foregoing an FA and using a less resource-intensive method to inform treatment (e.g., indirect and/or descriptive assessment). In contrast, if a client is engaging in self-injury daily in the form of banging their head against hard surfaces, clinicians should consider implementing an FA to optimize the likelihood of quickly developing an effective treatment and decreasing the risk for serious injury.^1^

## Ethics and Safety of FA

An important consideration when implementing an FA is the safety of the client. Some clinicians view FAs as ethically inappropriate (Roscoe et al., 2015a, 2015b). However, it is important to note that clients referred for an FA are those who are already engaging in challenging behavior in the natural environment, which may already pose a risk to the safety of themselves and others. For this reason, clinicians are ethically obligated to consider several factors before deciding an FA is appropriate for a given client, as well as during the assessment process to ensure any potential risks associated with conducting the FA are identified and addressed throughout. Before initiating an FA, clinicians should rule out medical or biological factors that may contribute to the challenging behavior (BACB ethics code 2.12), communicate effectively with stakeholders about the process (BACB ethics code 2.08), and involve clients and stakeholders in decision-making (BACB ethics code 2.09). For more detailed guidance on these ethical practices, Brown et al. (2022) offered valuable insights on incorporating family-centered care practices into behavior-analytic assessment and treatment.

Conducting an FA involves inherent risks because it creates conditions where challenging behavior might occur and may be reinforced. For instance, clients might injure themselves (e.g., self-injurious behavior), staff may be harmed by aggressive actions, or property could be damaged. These risks are significantly elevated if the FA is not conducted in a safe physical environment with competent staff and appropriate safety materials. Physical environment, staff competency, and safety materials are important considerations for FA adoption in clinical settings (Iwata & Dozier, 2008; Roscoe et al., 2015a, 2015b). Challenges associated with these variables should not prevent clinicians from using FAs to inform treatment. Appropriate safeguards can effectively mitigate risks, allowing for safer FAs with minimal or no injury (Betz & Fisher, 2011). For example, Kahng et al. (2015) found that the rate and severity of injuries during FA were generally lower than the rate and severity of injuries generally observed outside of the FA. These data suggest that when FAs are conducted with proper precautions—such as an increased staff-to-client ratio, protective equipment, immediate medical treatment for injuries, and individualized session-termination criteria—an FA may not necessarily be the primary source of injuries for individuals with severe challenging behavior.

### Pre-assessment Risk Evaluation

Despite the encouraging findings of Kahng et al. (2015), a recent survey by Deochand et al. (2020) revealed that 42% of BCBA/Ds agreed that FAs are inherently risky. This perception continues to be a barrier to adopting FAs in clinical settings. Additionally, almost all respondents expressed the need for a risk assessment tool with safety recommendations to guide decisions about when to conduct FAs. Deochand et al. collaborated with FA experts to develop an interactive risk assessment decision tool to address these concerns. This tool assesses risk as slight, moderate, substantial, or high across four primary domains identified and defined by Wiskirchen et al. (2017): clinical experience, behavior intensity, support staff, and environmental setting. The tool incorporates several considerations from the literature, such as recommending that clients undergo a medical screening by a healthcare provider to rule out potential medical causes for challenging behavior and identify necessary modifications to the FA (e.g., blocking to prevent removal of a gastronomy tube, session termination criteria, the potential impact of medication on challenging behavior). Additionally, the tool suggests evaluating the severity of destructive behavior and using this information to develop a clear decision-making plan to optimize safety for the client and staff during the FA. To this aim, clinicians can quickly administer the Destructive Behavior Severity Scale (Fuhrman et al., 2021; Irwin Helvey et al., 2024) with caregivers to gather information about the frequency, intensity, and topographies of challenging behavior. While research has yet to fully assess the tool’s effectiveness in guiding clinical decision-making about FAs, data from a recent pilot study suggested that the Functional Analysis Risk Assessment Tool may decrease the variability of risk ratings for novice behavior analysts. They also found that the use of the tool resulted in risk ratings that more closely matched the intended risk level of the vignettes for both expert and novice behavior analysts, suggesting the tool may be helpful in evaluating risks in FA (Schroeder et al., 2024). Free electronic copies of the decision-making tool are available online (Deochand et al., 2020).

### Mitigating Safety Risks During FA

Ensuring environmental safety during an FA involves making necessary modifications to the setting where the assessment will take place. This includes clearing the area of potentially dangerous objects such as pencils, sharp items, or breakable objects, and padding hard surfaces. When possible, padded floors and walls can further protect participants and staff from injuries associated with certain forms of self-injurious behavior or aggression. Programs that assess and treat severe challenging behavior often include additional safety and crisis management components. These may feature one-way observation mirrors made of tempered glass, two-way intercom systems, a paging system to alert the facility of potentially dangerous situations, and electromagnetic locks on treatment room doors (Fuhrman et al., 2021; Irwin Helvey et al., 2024). While these precautions may seem tailored to highly specialized clinical settings, clinicians in other applied settings should also prioritize safety during FAs. For example, napping mats for children can be used as temporary padding during FAs conducted outside of a clinical environment. Additionally, clinicians should ensure that building exits are locked and secured, the immediate environment is free of potential safety hazards, and emergency contacts are clearly outlined.

As staff conducting sessions are part of the FA environment, they should be appropriately dressed without dangling jewelry or loose-fitting clothes that could be targeted by challenging behavior. Additionally, staff with long hair should tie their hair back in case of hair pulling. Regarding protective equipment, there are options for both clients and staff, and specific types may be worn depending on the topography of challenging behavior. Although protective equipment may be necessary, clinicians should be aware that its use may mask functions in some cases (e.g., Le & Smith, 2002) or lead to the emergence of novel topographies of challenging behavior (e.g., Fisher et al., 1997; Parenteau et al., 2013). A complete list of protective equipment and environmental safety measures is beyond the scope of this paper. Readers are referred to Irwin Helvey et al. (2024) for considerations regarding the use and selection of protective equipment during FA. For the most part, the aforementioned recommendations to ensure environmental safety and personal protection have minimal costs and can be achieved in various settings (e.g., clinic, home, school).

There are several FA modifications that clinicians can make to optimize safety and minimize the risk of injury. Foremost, clinicians should arrange for brief periods of exposure to the establishing operation (EO) and continuous schedules of reinforcement. Reinforcing every instance of challenging behavior in the test conditions should immediately decrease the frequency and intensity of the challenging behavior (Hanley, 2012). Likewise, putative reinforcers should be arranged continuously and noncontingently in the control condition to decrease the EO for challenging behavior. These modifications and shortening session durations (e.g., 5 min) can aid in minimizing severe challenging behavior when it does occur during the FA. A second modification to safeguard against severe challenging behavior is to include multiple response topographies in the response class for which contingencies are programmed (Warner et al., 2020). Doing so may help minimize the occurrence of severe challenging behavior that is not reinforced during an FA of a separate topography. Although this modification can help safeguard against severe challenging behavior, it is important to note that FA outcomes are more likely to demonstrate challenging behavior is maintained by multiple reinforcement contingencies when multiple topographies are assessed (i.e., versus a single topography; Beavers & Iwata, 2011; Weber et al., 2024).

Finally, clinicians may also consider including a surrogate response. For example, Greer et al. (2024) used a square blue cushion during all parts of their experiment (including the pre-experimental FA), for which they programmed the same contingencies as target challenging behavior. Similar to this study, therapists can hold or place a colored cushion within close proximity to the client and, before each session, provide the instruction, “If you are upset, you can hit the pad,” and model the response. Importantly, surrogate responses should not be directly trained before the FA but can be made available as an alternative to challenging behavior during the FA to help promote the safety of the client and staff. This is because clinicians may inadvertently teach a new behavioral function if the surrogate response contacts reinforcement during pre-training before the FA. We encourage readers to reference Lambert and Boyle (2024) for a more comprehensive discussion on safety during FA implementation.

## How to Select FA Test Conditions

Regardless of the FA methodology used (e.g., traditional, trial-based), it is important to select appropriate test conditions. Failure to include relevant test conditions could have an untoward effect, such as a failure to identify a functional relationship. Inversely, including unnecessary test conditions can also have untoward effects (e.g., iatrogenic effects, described below). Clinicians should consider the following to identify relevant test conditions: (a) the outcomes of indirect and/or descriptive assessments, (b) the overall reported prevalence of the hypothesized reinforcer maintaining challenging behavior, and (c) potential untoward effects (e.g., iatrogenic effects) of including any given test condition. Each of these variables should be carefully considered when selecting FA test conditions.

### Outcomes from Indirect and Descriptive Assessments

Indirect and descriptive assessments are common tools in clinical settings to aid clinicians in identifying functions of challenging behavior (Oliver et al., 2015; Roscoe et al., 2015a, 2015b). Indirect assessments involve either open- or close-ended surveys of stakeholders. Descriptive assessments involve observing behavior in the client’s everyday environment and recording antecedents and consequences. Despite the popularity of indirect and descriptive assessments in clinical practice, neither of these approaches demonstrates high accuracy or reliability in determining the function(s) of challenging behavior (e.g., Call et al., 2024; Dracobly et al., 2018; Iwata et al., 2013; Zarcone et al., 2001). Although an in-depth analysis of the correspondence across FA and other functional behavior assessments is outside the scope of this paper, it is important to acknowledge their widespread use and provide our recommendation to use indirect and descriptive assessments to *inform* FA development. For example, descriptive assessments are commonly used to identify what test conditions to include in FA (Call et al., 2024; Contreras et al., 2023; Roscoe et al., 2015a, 2015b), the stimuli to include in selected test conditions (e.g., demands during escape test conditions; Fisher et al., 2016), and inform potential antecedents or consequences that may be specific to an individual (Hanley, 2012). Thus, we encourage clinicians to use indirect and descriptive assessments, regardless of the FA method employed, to inform the development of a more ecologically valid FA.

### Prevalence of Hypothesized Reinforcer

The relatively common occurrence of challenging behavior maintained by tangible, attention, escape from demands, and automatic reinforcement emphasizes the need for clinicians to consider incorporating these test conditions in FA. For example, Melanson and Fahmie (2023) conducted an updated review of published FAs, summarizing the last 40 years of data, and found more than 90% of FA outcomes indicated challenging behavior was maintained by either tangible, attention, escape from demands, and/or automatic reinforcement. Similar outcomes were obtained by Weber et al. (2024), who conducted the most extensive review of clinical FA outcomes to date (see also Hagopian et al., 2013). Collectively, decades of data on published and clinically obtained FA outcomes suggest clinicians should test for the most commonly identified sources of reinforcement prior to testing for sources of reinforcement less frequently identified (e.g., mand compliance; Owen et al., 2020). We encourage readers unfamiliar with designing these test conditions to review Dozier et al. (2022).

In the past decade, there has been an increasing trend in testing for multiple reinforcers within a single test condition (Melanson & Fahmie, 2023), often referred to as a synthesized contingency analysis (Hanley et al., 2014). The synthesized contingency analysis operates on the hypothesis that there is an interactive effect in which two or more reinforcers act on behavior to produce outcomes that would otherwise not be obtained with a single reinforcer (Fisher et al., 2016). For example, if an interactive effect between attention and tangible reinforcers is responsible for maintaining challenging behavior, one would find that testing for either of these reinforcers in isolation would either produce *no challenging behavior* or *lower levels of challenging behavior* relative to when the reinforcers are combined in a single test condition. The prevalence of interactive or synthesized FA outcomes is low (Fisher et al., 2016; Greer et al., 2020; McCabe et al., 2025; Weber et al., 2024). A recent study by McCabe et al. (2025) examined the evidence for interactive effects during and following a synthesized contingency analysis using published datasets. Despite conducting a robust evaluation by analyzing several different ways in which an interactive effect may be detected, the researchers found that synthesized contingency assessments rarely demonstrated interactive effects, leading the researchers to conclude that, in most cases, it is likely sufficient for clinicians to test for isolated reinforcement contingencies.

### Potential for Untoward Side Effects

Another important variable clinicians should consider when selecting FA test conditions is the potential for untoward side effects. One known untoward side effect of an FA is an iatrogenic effect, which is when a procedure causes a new symptom or condition (Retzlaff et al., 2020). During an FA, this occurs when a reinforcer is delivered contingent on challenging behavior, and increases the likelihood of the challenging behavior again in the future to access said reinforcer. Iatrogenic effects are problematic because challenging behavior now persists under novel stimulus conditions (that were not previously a referral concern) and may require multiple treatments or treatment components, which can be time- and resource-intensive. Iatrogenic effects have been found to occur in escape, attention, and tangible test conditions (Fernandez et al., 2024). However, researchers have reported the occurrence of iatrogenic effects most often during tangible test conditions (Brown et al., 2024a, 2024b; Kahng & Iwata, 1999; Rooker et al., 2011), leading to recommendations only to include a tangible test condition if indirect or descriptive assessments indicate challenging behavior may be maintained by access to tangibles (Melanson & Fahmie, 2023). It is also worth noting that automatically maintained challenging behavior may be particularly prone to iatrogenic effects. If challenging behavior that occurs independent of social consequences contacts a potent reinforcer in a social test condition (e.g., attention), the challenging behavior can become multiply maintained (i.e., the iatrogenic effect has occurred; e.g., Rooker et al., 2011; Shirley et al., 1999). Because of this, clinicians should screen for automatic reinforcement before conducting an FA that tests for social reinforcers to minimize the potential for iatrogenic effects (Querim et al., 2013).

Iatrogenic effects can occur during FAs that test for isolated reinforcement contingencies (e.g., Galiatsatos & Graff, 2003; Rooker et al., 2011) and FAs that synthesize reinforcement contingencies (e.g., Irwin Helvey & Van Camp, 2022; Retzlaff et al., 2020). However, research has shown that the likelihood of an iatrogenic effect is much greater during synthesized reinforcement test conditions (i.e., synthesized contingency analysis) relative to isolated reinforcement test conditions (e.g., Fernandez et al., 2024; Irwin Helvey & Van Camp, 2022). Because of this, we encourage clinicians to consider brevity in exposure to synthesized reinforcement test conditions and to only test for synthesized reinforcers if one or more isolated social reinforcers are not identified. In addition, we encourage clinicians to consider developing a plan to monitor for this untoward effect when using synthesized reinforcement test conditions (Ethics Code for Behavior Analysts, 2.13; please see Brown et al., 2024a, 2024b, for further discussion).

## How to Select an FA Methodology

There are several variables clinicians should consider when selecting which FA method to use with a client. Behavior analysts are ethically obligated to use an FA method with the strongest empirical evidence related to *the specific client, setting, and behavior* in question (BACB ethics code 2.13). Below, we outline six important considerations clinicians should consider when deciding which FA method to implement with a client. These considerations are empirical evidence, time, severity of challenging behavior, response topography, potential for an automatic reinforcement outcome, and low-rate behavior. Although each is an important consideration, this list is not exhaustive, and additional considerations are necessary (e.g., caregiver preference).

### Consideration #1: Empirical Evidence

The goal of any FA is to identify the reinforcer(s) maintaining challenging behavior and use this information to inform the development of a function-based intervention (Hagopian et al., 2013; Vollmer et al., 2020). Inaccurate or contraindicated FAs can have several deleterious effects, such as failing to identify a potential function (a false-negative outcome), identifying an *incorrect* potential function (a false-positive outcome), or teaching a new function that was not previously there (an iatrogenic effect), all potentially leading to the development of a less effective treatment (Holehan et al., 2020; Iwata et al., 1994/1982; Irwin Helvey & Van Camp, 2022; Retzlaff et al., 2020). Given this, clinicians must select the FA method with the most empirical evidence or rigor. Empirical evidence does not equate to the number of publications on a given FA method (e.g., trial-based, multielement) or its reported use. Instead, empirical evidence refers to data on how *accurate* FA outcomes are for a given method.

When considering the empirical evidence or accuracy of an FA, there are several key terms of which readers should be aware (see Table 1 for a visual depiction of these terms and definitions). These include (a) sensitivity, (b) specificity, (c) discriminant validity, and (d) predictive validity (Tiger & Effertz, 2021). In brief, *sensitivity* refers to the ability of an FA to detect and rule in a potential function accurately. For example, if challenging behavior is maintained by access to tangibles, a sensitive FA should demonstrate tangible reinforcement as an outcome. *Specificity* refers to the ability of an FA to rule out potential functions accurately. Using the same example above, if challenging behavior is maintained by access to tangibles, an FA with good specificity should be able to “rule out” stimuli that do not function as reinforcers (e.g., attention, escape from demands). An assessment low on either sensitivity or specificity is limited in its clinical usefulness. *Discriminant validity* relates to how well an FA can distinguish or differentiate separate functional classes of reinforcement. That is, an FA with good discriminant validity can identify the specific functional classes of reinforcement that maintain challenging behavior. This is essential, especially in cases where an individual might engage in different topographies of challenging behavior, each maintained by a different function, or where challenging behavior serves multiple functions. Finally, *predictive validity* refers to the probability that the results of an FA will lead to desirable treatment outcomes. Again, using the same example, if the FA that identified a tangible function has good predictive validity, then the treatment derived from the outcomes of that FA should lead to reductions in challenging behavior and increases in desirable behavior (e.g., a communication response).

It is worth noting that not all FA methods demonstrate high sensitivity, specificity, discriminative validity, and predictive validity. For example, the synthesized contingency analysis has repeatedly shown poor sensitivity and specificity but high predictive validity (see Tiger & Effertz, 2021, for a discussion; see Call et al., 2024, for an example). As such, we encourage clinicians to become familiar with each FA methodology’s relative strengths and weaknesses and to routinely select the most empirically accurate FA method available while considering other important individual case variables. Table 2 lists FA methods from most to least efficacious based on existing literature (top-to-bottom; see Rahaman et al., 2024, for a full review of correspondence across FA methods).

**Table 2:**
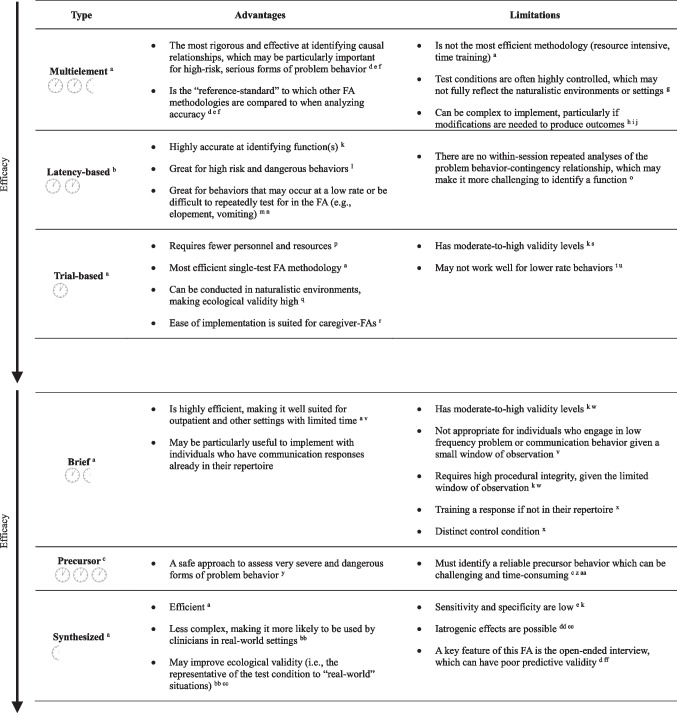
Advantages and limitations of functional analysis methodologies

Clock icons indicate the estimated time to complete each methodology so that one full clock icon indicates about an hour of time for completion. Sources for average time completion are as follows. ^a^ Saini et al. (2020). ^b^ Griffith et al. (2021). ^c^ Borrero and Borrero (2008). ^d^ Fisher et al. (2016). ^e^ Tiger and Effertz (2021). ^f^ Call et al. (2024). ^g^ Hanley et al. (2003). ^h^ Hagopian et al. (2013). ^i^ Hanley (2012). ^j^ Weber et al. (2024). ^k^ Rahaman et al. (2024). ^l^ Neidert et al. (2013). ^m^ Thomason-Sassi et al. (2011). ^n^ Traub and Vollmer (2019). ^o^ Vollmer et al. (1993). ^p^ Kodak et al. (2013). ^q^ Sigafoos and Saggers (1995). ^r^ Bloom et al. (2013). ^s^ Rispoli et al. (2013). ^t^ Bloom et al. (2011). ^u^ Dowdy et al. (2021). ^v^ Derby et al. (1992). ^w^ Call et al. (2013). ^x^ Northup et al. (1991). ^y^ Smith and Churchill (2002). ^z^ Fritz et al. (2013). ^aa^ Lydon et al. (2012). ^bb^ Hanley et al. (2014). ^cc^ Slaton and Hanley (2018). ^dd^ Irwin Helvey and Van Camp (2022). ^ee^ Retzlaff et al. (2020). ^ff^ Greer et al. (2020)

### Consideration #2: Time

Another important consideration when selecting an FA method is the total time available to conduct the FA. Efficiency has been a longstanding barrier to FAs being conducted in clinical settings (Caldwell et al., 2023; Hanley, 2012; Iwata & Dozier, 2008; Oliver et al., 2015; Roscoe et al., 2015a, 2015b). A recent survey highlighted key time-related challenges, including limitations within work schedules, insufficient time for developing an FA, completing associated administrative tasks, and the time required to obtain results (Caldwell et al., 2023). For clinicians for whom time is a significant barrier to FA implementation, we encourage them to see Caldwell et al. (2023) for an in-depth discussion on system-level solutions on how to mitigate time barriers. Below, we outline strategies for addressing time-related challenges within FAs.

First, we encourage clinicians to become familiar with FA modifications that can decrease the time needed to obtain results. For example, common modifications that can save time include limiting individual test conditions to those informed by indirect and/or descriptive assessments, shortening session durations to 5 min, and including multiple topographies of challenging behavior (Melanson & Fahmie, 2023; Warner et al., 2020). For example, rather than conducting a separate FA on aggression and property destruction, both responses can be consequated during one FA. Suppose there is uncertainty regarding whether multiple topographies function as members of the same response class. In that case, another approach is to focus on the primary topography of concern while concurrently gathering data on other topographies to screen for potential functions (Bell & Fahmie, 2018). Second, clinicians should select an FA method to overcome time constraints, such as the brief (Derby et al., 1992; Northup et al., 1991) or trial-based (Bloom et al., 2011; Sigafoos & Saggers, 1995) FA. Third, clinicians should use structured criteria for session termination, as this has been shown to decrease the mean number of sessions needed to identify a function. For example, Saini et al. (2020) systematically reviewed the literature to compare the efficiency of common FA methods. They found that using ongoing visual inspection, a type of structured decision-making criteria, reduced the mean number of sessions required to identify a function across FA methods by 4% to 35%, depending on the FA method. We provide guidelines for data interpretation in the section below analyzing FA outcomes. Finally, clinicians may consider conducting within-session analyses of responding to help facilitate timely assessment termination (Roane et al., 1999). Within-session analysis involves calculating rates of challenging behavior during the reinforcement intervals of the test condition (EO absent) compared to rates of challenging behavior during the remaining session time (EO present). If the stimulus is a functional reinforcer, its delivery should serve as an abolishing operation, reducing the occurrence of the challenging behavior during the reinforcement interval. For example, if access to adult attention is a functional reinforcer, rates of challenging behavior should be relatively low when adult attention is provided (i.e., EO absent) compared to relatively high rates when adult attention is withheld (i.e., EO present) during a test for social positive reinforcement. By analyzing within-session rates of challenging behavior, clinicians may quickly observe differentiation between establishing-operation-present intervals and establishing-operation-absent intervals and, therefore, conduct fewer total sessions of the FA.

In addition to the aforementioned considerations, clinicians can increase the efficiency of FA by using an evidence-based model on how to progress from brief to extended FA methods (e.g., Henry et al., 2021; Slanzi et al., 2022). For example, Henry et al. (2021) evaluated a five-phase progression that began with several sessions of no-interaction or alone conditions to screen for automatic reinforcement. Subsequent phases included a brief FA, within-session analysis, and isolated contingency FAs with adjustments for enhanced differentiation, extended alone/no interaction, and participant-specific design modifications. By the end of the final phase, a function was identified for 100% of participants. Notably, the initial series of no-interaction or alone sessions predicted automatic reinforcement for all participants whose challenging behavior persisted in this phase. This finding is consistent with other studies using a screener for automatic reinforcement (Querim et al., 2013; Slanzi et al., 2022) and suggests clinicians begin with a screener for automatic reinforcement and proceed immediately to treatment if the response persists. Overall, decision-making models may help minimize assessment time while encouraging a more rigorous demonstration of functional control over challenging behavior through systematic modification of the analysis.

As detailed above, several time-saving considerations can increase the efficiency of conducting FA. Importantly, findings from a recent systematic literature review found that there are multiple FA methods clinicians can use to test single or multiple functions within a 90-min appointment (Saini et al., 2020). As such, FA methodology refinements have made it such that time is no longer a significant barrier to FA implementation in clinical settings. In Table 2, each FA method has an efficiency indicator. We encourage clinicians to use this information as a reference when considering time as a variable when selecting an FA method.

### Consideration #3: Severity of Challenging Behavior

An important consideration when selecting an FA method is the severity of challenging behavior. A common perception among clinicians is that FA poses safety concerns, mainly when working with individuals who engage in severe challenging behavior. Caldwell et al. (2023) found that 35% of Board Certified Behavior Analysts (BCBA/Ds) reported a belief that FAs were unsafe for addressing dangerous behaviors, and around 5% of respondents from Roscoe et al., (2015a, 2015b) reported feeling unsafe conducting an FA. Although there may be instances where challenging behavior is exceptionally severe, resulting in unmanageable risks of injury to oneself or others, we advocate for clinicians to assess the relative risks and benefits on a case-by-case basis (see Kahng et al., 2015 for related discourse). Opting not to conduct an FA may inadvertently create other potential risks, as outlined above. Thus, in lieu of an exceptional case situation with unmanageable risk, clinicians should opt to conduct an FA and select a method that allows for safe assessment.

Researchers have explored several FA methods for assessing severe challenging behavior. A primary consideration is to select an efficient FA method that reduces total assessment time (e.g., brief, latency-based, or trial-based). Perhaps the precursor FA is the most relevant to assessing severe challenging behavior (Smith & Churchill, 2002). Regardless of the FA method selected, clinicians must optimize client safety by using as many environmental and procedural protections as possible, following the recommendations outlined in the current paper's Ethics and Safety section.

### Consideration #4: Response Topography

In some cases, the nature of specific response topographies and the reactions from others when they occur may dictate the need for certain FA methods relative to others. For example, elopement poses a serious life-threatening risk that often results in extreme reactions from caregivers and others who intervene by retrieving the individual who has eloped for apparent safety reasons. However, in an FA, providing attention in the form of retrieval can confound the results in that it resets the EO. To minimize the confound of providing attention when repeatedly retrieving a client following an elopement, researchers have suggested the use of a latency-based FA (Kamlowsky et al., 2021; Neidert et al., 2013; Traub & Vollmer, 2019).

Pica is another response topography that could be life-threatening and requires unique consideration when selecting an FA method. A latency-based FA may be appropriate to reduce repeated ingestion of potentially dangerous items and opportunities to access unprogrammed consequences. Alternatively, clinicians could consider a more rigorous method, such as the traditional FA, and “bait” the environment with materials that would not be dangerous if ingested. For example, Piazza et al. (1998) evaluated pica using a traditional FA and baited session rooms with food items that medical professionals cleared as safe for mouthing or consumption (e.g., pieces of rice paper, uncooked beans and pasta, rice sticks). Therapists then blocked attempted pica of other non-baited items. Similarly, Morris et al. (2021) conducted the only known study that used baited materials during an FA of pica outside highly controlled settings. For the in-home FA, researchers cleared the leisure room of items that could be dangerous if ingested (e.g., pencils, fabric-covered furniture). However, clearing all hazardous materials from a room may be impractical in certain situations, such as common family rooms or classrooms. In such cases, clinicians are advised to use the latency-based FA to assess pica.

### Consideration #5: Potential Automatic Reinforcement Function

Clinicians also need to consider the potential of challenging behavior being maintained by automatic reinforcement. Although it is valuable to include a screener for automatic reinforcement routinely, we recognize clinicians may not always be able to include automatic reinforcement screeners in every case. In these circumstances, we encourage clinicians to attend to case variables that may suggest the need for including an automatic reinforcement screener. For example, clinicians should consider including an automatic screener if indirect or descriptive assessments suggest one or more topographies of challenging behavior may be maintained by automatic reinforcement. Clinicians should also include a screener for automatic reinforcement for cases in which the individual engages in stereotypy, pica, or self-injurious behavior, given that research has found these topographies are frequently found to be maintained by automatic sources of reinforcement (Melanson & Fahmie, 2023). Conversely, FA outcomes for aggression, noncompliance, elopement, vocalization, and property destruction less frequently suggest automatic reinforcement. As such, if the primary referral concerns involve self-injurious behavior, stereotypy, or pica, an efficient and practical approach would be to conduct a screener for automatic reinforcement (Henry et al., 2021; Querim et al., 2013; Slanzi et al., 2022) prior to conducting a more thorough FA (if needed). For other topographies of challenging behavior, clinicians might opt to begin the FA without screening for automatic reinforcement when facing time and resource constraints, given the lower likelihood of an automatic reinforcement function.

### Consideration #6: Low-Rate Behavior

A final consideration when selecting an FA method is the reported rate of challenging behavior. Challenging behavior that occurs at a low (or zero) rate can result in FA outcomes that are inconclusive or undifferentiated. Although not commonly reported in the literature, large *n* consecutive controlled case reviews of FA outcomes have found undifferentiated outcomes occur with some relative frequency in clinical settings. For example, Hagopian et al. (2013) found that 6.7% of cases resulted in undifferentiated outcomes. A recent study by Weber et al. (2024) found that 17.9% of cases resulted in undifferentiated outcomes. Inconclusive FA results may be the result of not including the relevant antecedent or consequences (Roscoe et al., 2015b; Schlichenmeyer et al., 2013), not programming a long enough window of measurement (Kahng et al., 2001), or reactivity (Rooker et al., 2015).

#### Did I Include the Relevant Test Conditions?

Suppose one or more of the commonly identified reinforcers (e.g., escape, attention, tangible) are not found to maintain challenging behavior in the initial FA. In that case, clinicians may want to consider if the relevant test conditions were programmed. Identifying potential idiosyncratic reinforcers maintaining challenging behavior can be achieved by conducting additional indirect and descriptive assessments (Roscoe et al., 2015a, 2015b). For example, Roscoe et al., (2015a, 2015b) provide a close-ended list of questions clinicians may use to identify potential idiosyncratic events. Similarly, Hanley (2012) provides an open-ended list of questions clinicians can ask caregivers to help identify participant-specific variables that may be functionally related to challenging behavior. In addition, clinicians may also consider moving to a synthesized contingency assessment (Hanley et al., 2014) to examine if challenging behavior is maintained by interactive control of multiple social contingencies.

#### Do I Have a Long Enough Observation Window?

Low-rate behavior may also result from not having a long enough period to observe the target behavior(s). One strategy to address low-rate behavior is to start FA sessions contingent on the occurrence of challenging behavior. For example, Tarbox et al. (2004) initiated sessions contingent on at least one instance of challenging behavior and conducted at least one series of each test and control condition once sessions were initiated. Researchers continued with additional series as long as the criterion of at least one instance of challenging behavior was met between each series. A second strategy to identify functions for low-rate behavior is to increase session duration. For example, Wallace and Iwata (1999) compared data sets from the last 5 min, 10 min, and 15 min of FAs. When discrepancies were observed, they were due to increased response rates later in the session. Other researchers have identified functions of challenging behavior by conducting day-long FAs (i.e., 8 h; Kahng et al., 2001). A third strategy that may be valuable in cases with low-rate behaviors is to have caregivers implement the FA (see Brown et al., 2024a, 2024b, for more information on caregiver-implemented FAs).

#### Am I Observing Reactivity?

Participants responding during an FA may be suppressed owing to reactivity to environmental variables that do not reflect those in the referral context (Rooker et al., 2015). Clinicians can address this by rearranging the environment to be more naturalistic. For example, if no responding is observed in a clinic room, clinicians might introduce items similar to those in the participant’s natural environment. Alternatively, they could consider changing the location, such as conducting the FA in the participant’s home. Beyond the physical environment, clinicians should use indirect and descriptive assessments to inform the development of FA test conditions such that these emulate interactions that the client has with caregivers and other change agents (Rooker et al., 2015).

Participants who engage in covert challenging behavior may also be reactive to the presence of observers. For example, Simmons et al. (2019) used a latency FA to evaluate food stealing. They found more frequent and quicker responding during the alone condition, with longer latencies during the ignore condition and tests for socially mediated reinforcement. These results suggest that food stealing was covert and unlikely to occur in the presence of caregivers or other observers. In such cases, clinicians may need to use more discrete observation and data collection methods. To achieve this, data collectors could observe from a one-way observation mirror, use video-recording equipment, or take data based on permanent products of the challenging behavior.

Finally, participants may be reactive to the use of protective equipment for staff (e.g., Hood et al., 2019; Saini et al., 2015) or themselves (e.g., Le & Smith, 2002; Moore et al., 2004). If it is safe, clinicians could consider conducting FAs with and without the presence of protective equipment, ensuring participant and staff safety, to evaluate its impact. If it is deemed unsafe to conduct an FA without protective equipment, clinicians could make the equipment less noticeable, such as wearing a compression shirt to cover the chest and arm pads (Irwin Helvey et al., 2024).

## FA Methodology: Relative Strengths and Weaknesses

In the current paper, we focused on summarizing various FA methods instead of experimental designs for two reasons. First, readers can find a comprehensive and helpful overview of experimental designs used in FAs and how each achieves experimental control in Iwata and Dozier (2008). Second, although several comprehensive texts discuss FA methodologies (e.g., Dozier et al., 2022; Falligant et al., 2023), there is currently no tutorial for clinicians on *what variables to consider when selecting an FA methodology for a given client.* As such, we provide a brief overview of each FA methodology and its relative strengths and weaknesses. Note that the information in this section is not meant to be a comprehensive review, and we encourage readers unfamiliar with FA methodology to review previously referenced texts for this information. Table 2 presents a concise review of the information covered in this section.

Some clinical practices can promote the efficacy and/or efficiency of FA methods. For FA methods that use an experimental design with rapidly changing test and control conditions (e.g., multielement, pairwise), clinicians should program unique discriminative stimuli (e.g., therapist shirt color, colored poster board in the room) to facilitate the client discriminating between the different programmed reinforcement contingencies (Hammond et al., 2013). Clinicians should also consider implementing a fixed order of test conditions when testing for multiple isolated reinforcers (ignore, attention, tangible, play, and demand), given that this has been found to increase the efficacy and efficiency of FA (Hammond et al., 2013). Another reason to incorporate a fixed order of conditions is that it can help accommodate influences of establishing operations on responding. However, clinicians should be aware that using a fixed order of conditions has the potential to create *sequence effects*. The *overall order of conditions* can ultimately impact responding across conditions. If a clinician identifies potential sequence effects, then it might be helpful to incorporate a random order of test conditions. We encourage readers to review Dozier et al. (2022) for a more complete discussion of best practices in FA.

### Traditional

The traditional FA is the most widely recognized (Iwata 1982/1994) and involves the rapid alternation between multiple test conditions and one control condition in a multielement design. In a traditional FA, therapists collect repeated measures of behavior within a session (Iwata & Dozier, 2008). The traditional FA is often the reference standard to which other FA methodologies are compared (Call et al., 2024; Fisher et al., 2016; Tiger & Effertz, 2021), likely due to the large body of empirical research supporting its high accuracy in identifying causal relationships (challenging behavior → reinforcer). Because of this, the traditional FA is well-suited for complex and severe forms of challenging behavior. That said, the traditional FA may not be the most efficient methodology and can be resource-intensive in terms of time (Saini et al., 2020) and skills required of the therapist, particularly if modifications are needed to produce outcomes (Hagopian et al., 2013; Hanley, 2012; Weber et al., 2024). Finally, the rigor of the traditional FA often results in test conditions that are highly controlled and may not fully reflect naturalistic environments or settings, which may be challenging to contrive in everyday environments (e.g., home, school; Hanley et al., 2003).

### Brief

Despite its efficiency and efficacy, the brief FA (Northup et al., 1991) remains one of the least used FA methodologies (Melanson & Fahmie, 2023). The brief FA takes about 90 min to complete and consists of a series of test conditions and contingency reversal conditions (control). A primary advantage of the brief FA is its efficiency compared to other FA methods (Saini et al., 2020), making it well-suited for outpatient and other settings in which time is limited (Derby et al., 1992). Additionally, the brief FA may be a particularly useful FA to implement with individuals who already have communication responses in their repertoire.

There are some disadvantages to the brief FA clinicians should be aware of. Because of the limited time used to conduct the brief FA, it should not be used with individuals who engage in low rates of challenging behavior (Derby et al., 1992), as the shortened time window is not optimal for observing low-frequency behavior. Further, owing to the limited time window, high procedural integrity is required when conducting the brief FA, as this method has been shown to have lower levels of correspondence relative to more lengthy FA methods (Call et al., 2013; Rahaman et al., 2024). Finally, if the client does not already have a communication response in their repertoire, then clinicians would need to train this response before using this FA method.

### Trial-Based

The trial-based FA consists of trials with two segments (control and test) and is the most efficient single-test FA methodology, with the average time to completion being 60 min (Saini et al., 2020). Its efficiency and need for few resources (e.g., personnel; Kodak et al., 2013) makes this FA method highly suited for less controlled settings (e.g., homes, schools) in which clinicians or caregivers could easily implement the segments during a client’s routine (e.g., during instructional time at school; Bloom et al., 2013; Sigafoos & Saggers, 1995). The trial-based FA may not be appropriate for low rates of challenging behavior that are unlikely to be evoked with shorter programmed test conditions (Bloom et al., 2011; Dowdy et al., 2021). Finally, previous research has shown that the accuracy of trial-based FAs in identifying the function(s) of challenging behavior can range from moderate to high (Rahaman et al., 2024; Rispoli et al., 2013).

### Synthesized Contingency Analysis

The synthesized contingency analysis consists of a single test condition and control condition that alternate in a pairwise design (Hanley et al., 2014). This FA method is unique because it is the only method that combines potential reinforcers from the onset of analysis, whereas all other FA methods test isolated reinforcers. Owing to combining multiple reinforcers in a single test condition, this method is often more efficient relative to some other methods (Saini et al., 2020). It also may be considered less complex, increasing its use in clinical settings (Hanley et al., 2014). Finally, given the flexible nature of designing test conditions in this approach, it may improve ecological validity or how akin the test conditions are to “real-world” contexts (Hanley et al., 2014; Slaton & Hanley, 2018).

There are some disadvantages to the synthesized contingency analysis. First, as previously described, the synthesized contingency analysis is more likely to produce iatrogenic effects due to programming multiple reinforcers in a single contingency, relative to other FA methods that test isolated reinforcement contingencies (Irwin Helvey & Van Camp, 2022; Retzlaff et al., 2020). A second limitation of the synthesized contingency analysis is that false-negative (i.e., missed a function) and false-positive outcomes (i.e., identified a function that was not actually present) are considerably higher with this method, again due to programming multiple reinforcers in a single test condition. For example, Fisher et al. (2016) conducted a synthesized contingency analysis with five clients and found false-positive outcomes for four of the five clients (i.e., 80% of their clinical sample). Greer et al. (2020) extended this line of inquiry. They found that the synthesized contingency analysis resulted in false positive outcomes for six of nine clients who engaged in challenging behavior during the FA (i.e., 66.6% of their sample). Thus, the risk of false-positive and false-negative outcomes is substantially higher in the synthesized contingency assessment relative to the other FA methods (Rahaman et al., 2024; Tiger & Effertz, 2021). False-positive outcomes are problematic in that they could result in the inclusion of unnecessary treatment components and/or an increased likelihood of poor caregiver integrity or adherence in naturalistic settings due to a more complex treatment. False-negative outcomes may result in the re-emergence of challenging behavior that has not been functionally treated or in clinicians’ use of more restrictive procedures to obtain clinically significant reductions (Rahaman et al., 2024). A full discussion on the accuracy and safety of the synthesized functional analysis is outside the scope of this paper. We encourage readers who are unfamiliar with the strengths and limitations of this approach or the conditions under which clinicians should or should not consider this FA method to review Kranak and Briggs (2025).

### Latency-Based

The latency-based FA is unique because the first instance of challenging behavior in the test condition produces access to the reinforcer and terminates the session (e.g., Lambert et al., 2017). Because of this, the latency-based FA is uniquely suited for cases with extremely dangerous behaviors that pose a high risk of injury to oneself or others (Thomason-Sassi et al., 2011). Latency-based FAs are often highly accurate at identifying the function(s) of challenging behavior (Rahaman et al., 2024) and able to identify function quicker, with fewer instances of challenging behavior, relative to other forms of FA (Thomason-Sassi et al., 2011; Traub & Vollmer, 2019). Finally, the latency-based FA is well-suited to examine behaviors that are low-rate or difficult to repeatedly test for in a single test condition (e.g., elopement; Neidert et al., 2013). However, it can be challenging to identify a function of behavior using this method because there are no repeated measures of behavior within a single session (Vollmer et al., 1993).

### Precursor

The precursor FA is useful when challenging behavior is too severe to allow it to occur safely, such as severe aggression or self-injury (Smith & Churchill, 2002). The precursor FA involves programming contingencies for behaviors that have been found to precede challenging behavior reliably. For example, specific precursors may be part of a response chain, such as picking up a toy before throwing it. Precursor behaviors are typically less dangerous than challenging behavior, so the idea is that delivering reinforcement for precursors should keep challenging behavior low while also enabling clinicians to identify its function. Precursor behaviors may be identified through informal observation, often done by caregivers, or through more objective and rigorous approaches (Heath Jr. & Smith, 2019). Once a reliable precursor behavior has been identified, the precursor produces the programmed consequence instead of challenging behavior to identify maintaining reinforcers.

Conclusions about behavioral function may be inaccurate if precursors do not belong to the same response class as the target challenging behavior(s). Because of this, identifying reliable precursors to challenging behavior is very important. However, methods to examine the predictiveness of suspected precursors for challenging behavior can be time-consuming (conditional probability analysis; e.g., Borrero & Borrero, 2008; Fritz et al., 2013; Lydon et al., 2012). As such, the precursor FA should be used to identify variables that maintain severe forms of challenging behavior in which other FA methodologies cannot be used (Heath Jr. & Smith, 2019).

## Interpreting FA Outcomes

An FA is often said to be complete once differentiation is achieved and a functional relation is identified. In other words, an FA should continue (and be modified if necessary) until the data obtained from at least one test condition separates itself from the control condition when graphed and analyzed (see Hagopian et al., 2013). Differentiation is often identified through visual analysis (e.g., Betz & Fisher, 2011), in which clinicians inspect all of the requisite data paths in terms of trend, level, variability, and stability to determine which, if any, test conditions are differentiated from the control condition.

The extent to which one can accurately and reliably interpret FA outcomes through visual analysis heavily relies on the training and expertise of the person interpreting the results (Danov & Symons, 2008; Kahng et al., 2001). Although all BCBAs likely receive some training on how to analyze and interpret FA data in their graduate coursework, mastery of this skill requires the ability to make fine, and often times nuanced, discriminations (see Betz & Fisher, 2011; Roane et al., 2013). As such, researchers have developed decision-making tools to aid in and support visual analysis of FA outcomes (see Dowdy et al., 2022, for a review). One validated tool is the structured criteria method.^2^ Briefly, the structured criteria method involves (a) overlaying two criterion lines—the upper criterion and lower criterion line—onto an FA graph to represent the range where the majority of data points in the control condition fall, (b) counting the number of data points in each test condition that fall both above and below each criterion line, and (c) determining if there is a sufficient level of differentiation between a given test condition and the control condition. Note that several additional decision-making rules are applied related to various aspects of the data paths (e.g., rate, variability). Notably, the structured criteria method can be applied after FA completion (often called post-hoc visual inspection) or in *real-time* during an FA (often called ongoing visual inspection; see Saini et al., 2018). Ongoing visual inspection can be very amenable to clinical settings in which the FA results must be analyzed concurrently with the conducting of the FA. We encourage readers unfamiliar with ongoing visual inspection to see Szikszai et al. (2023).

Two patterns occur in an FA when challenging behaviors are maintained by sources of automatic reinforcement (e.g., Virues-Ortega et al., 2022). First, challenging behavior may occur at a high and steady rate in the no-interaction or alone condition, while little to no responding occurs in all other FA conditions. Thus, the no-interaction or alone condition is differentiated from the control condition, and very little challenging behavior occurred in test conditions that involved socially mediated reinforcement (which may have competed with sources of automatic reinforcement). Second, the outcomes of the FA are entirely undifferentiated because challenging behavior occurred at high and steady rates across all test conditions and the control condition. If either of these patterns of responding is observed, the behavior being assessed may have an automatic reinforcement function. Alternatively, clinicians might conduct either a pairwise comparison with a no-interaction or alone condition that alternates with the control condition or conduct repeated alone sessions to verify that challenging behavior persists in the absence of social consequences.

## Concluding Remarks

Since its inception, FA has been a cornerstone in the assessment and treatment of severe challenging behavior, with continued widespread acknowledgment of its importance in accurately identifying the precise reinforcer(s) maintaining challenging behavior (e.g., Call et al., 2024; Greer et al., 2020; Tiger & Effertz, 2021). The current paper is designed to support clinician decision-making by outlining the relative strengths and weaknesses of the FA methodologies and key variables that clinicians should consider when selecting an FA method for a given client. Our goal was to aggregate available research on FA efficacy, safety, efficiency, and practicality to support clinicians new to implementing FAs in clinical practice on *when* and *under what conditions* to include specific FA test conditions and select a given FA methodology for a client. Nonetheless, these suggestions should be considered in light of the continually evolving and expanding FA research.

## Data Availability

Data sharing is not applicable to this article as no datasets were generated or analyzed during the current study.

## Footnotes

1 These examples are provided for illustrative purposes only. Clinicians need to exercise clinical judgement to determine if an FA should be conducted on the basis of each client’s clinical presentation among several other relevant variables (e.g., client history, caregiver preference).

2 Hagopian et al. (1997) and modified by Fisher et al. (2003) and Roane et al. (2013).
